# Supplementary material for: Above-below surface interactions mediate effects of seagrass disturbance on meiobenthic diversity, nematode and polychaete trophic structure
Source: Commun Biol. 2019 Oct 4;2:362. doi: 10.1038/s42003-019-0610-4 (PMC6778119; doi:10.1038/s42003-019-0610-4)
Supplement: Supplementary file 1 — Supplemental Information [file 42003_2019_610_MOESM1_ESM.pdf]

Supplemental Information

Supplementary Figures

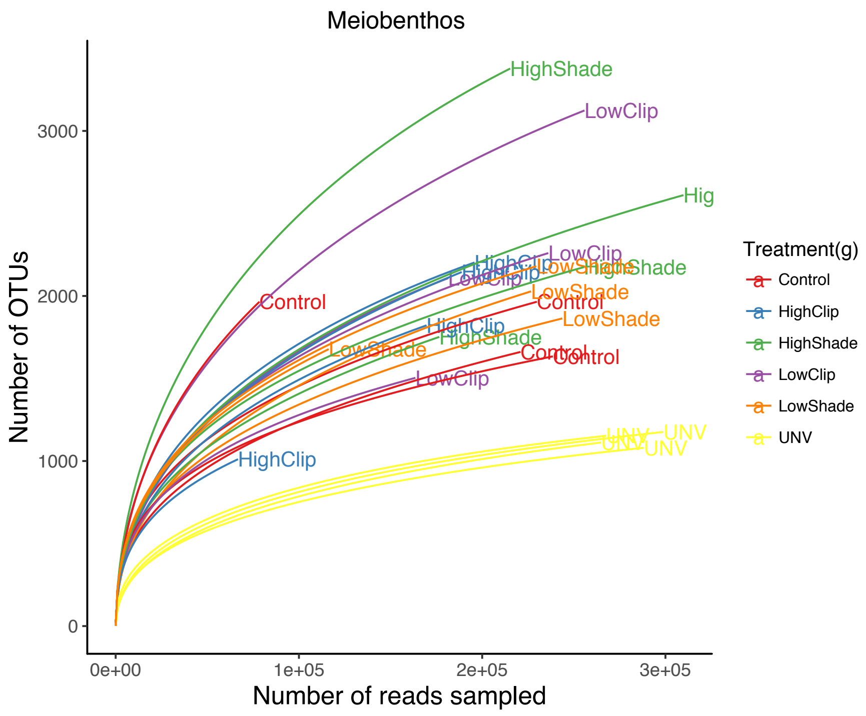

Supplementary Figure 1- Accumulation curve of number of meiobenthic OTUs vs number of sequencing reads in each sample.

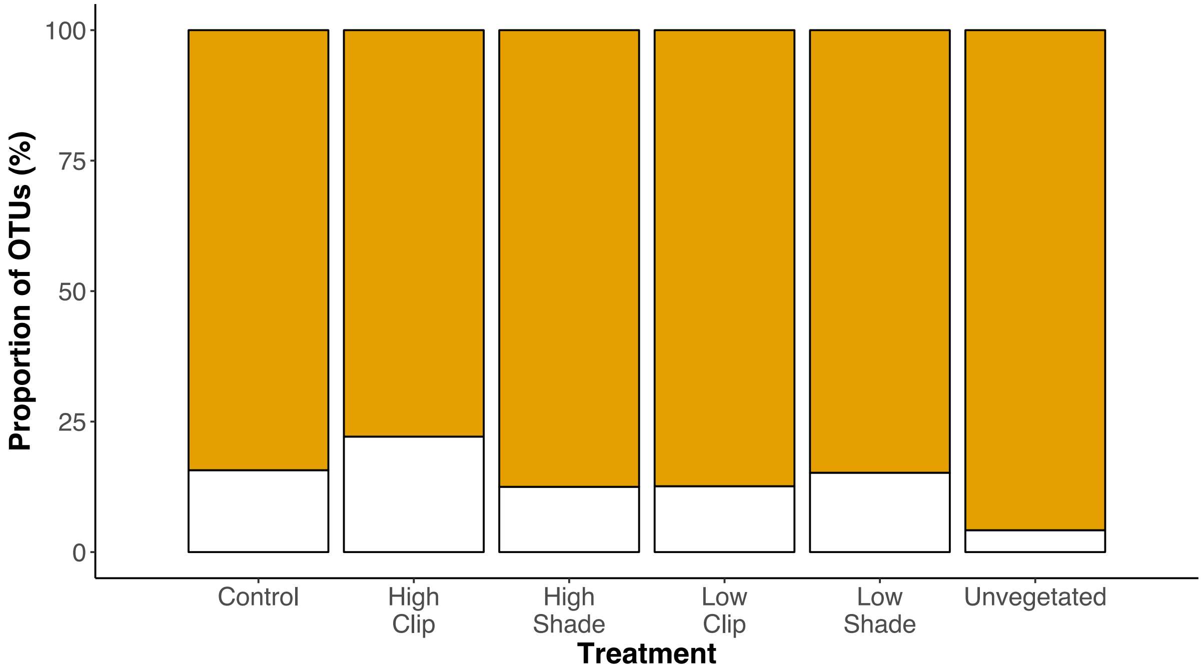

Supplementary Figure 2- Proportion of Metazoan (full bar) vs Non- Metazoan Eukaryotes (empty bars) in the different treatments.

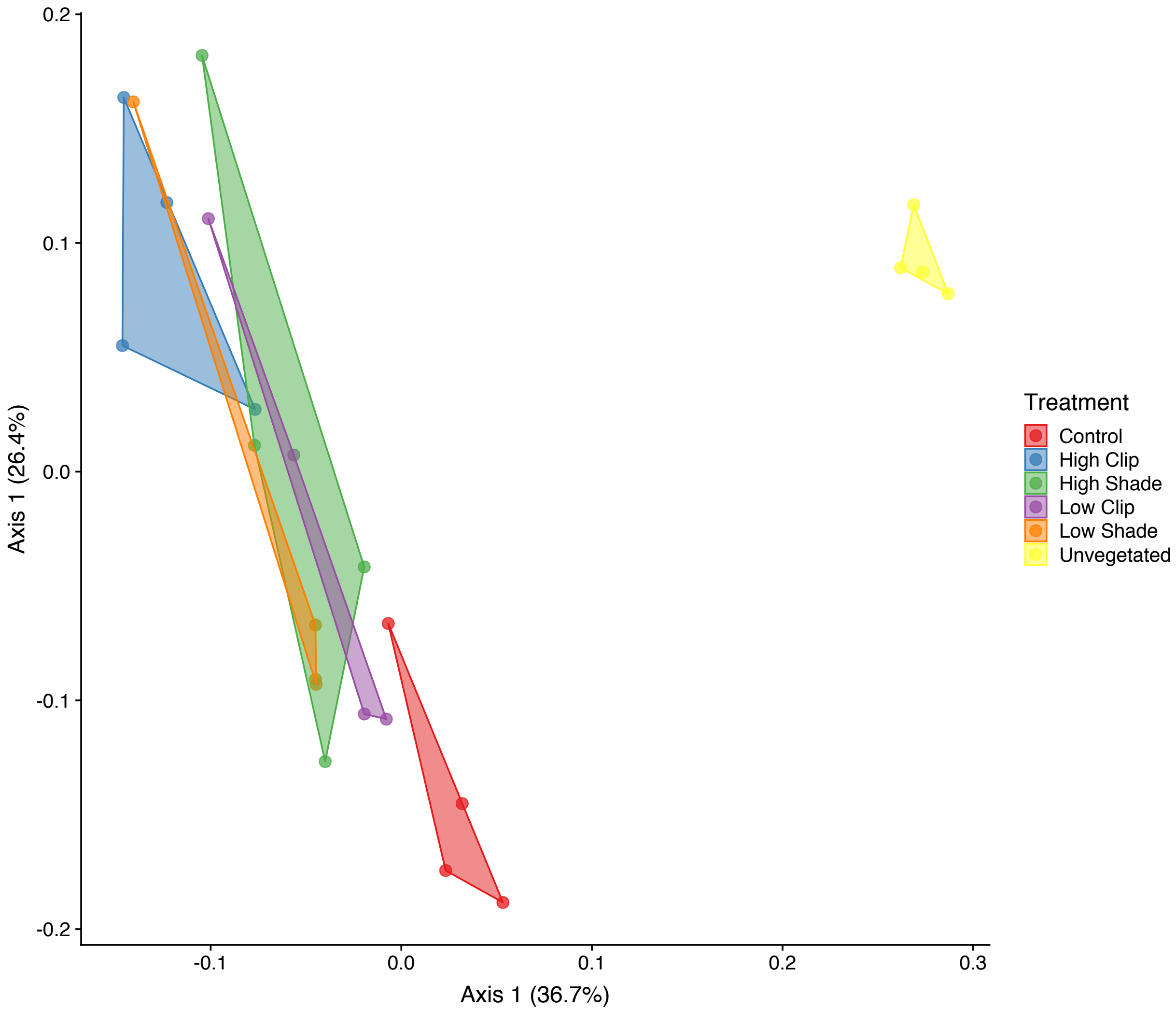

Supplementary Figure 3- Principal Coordinates Analysis (PCoA) based on normalized OTU matrix for meiobenthos using Unifrac distance dissimilarities. Different colours represent the groupings of the different treatments.

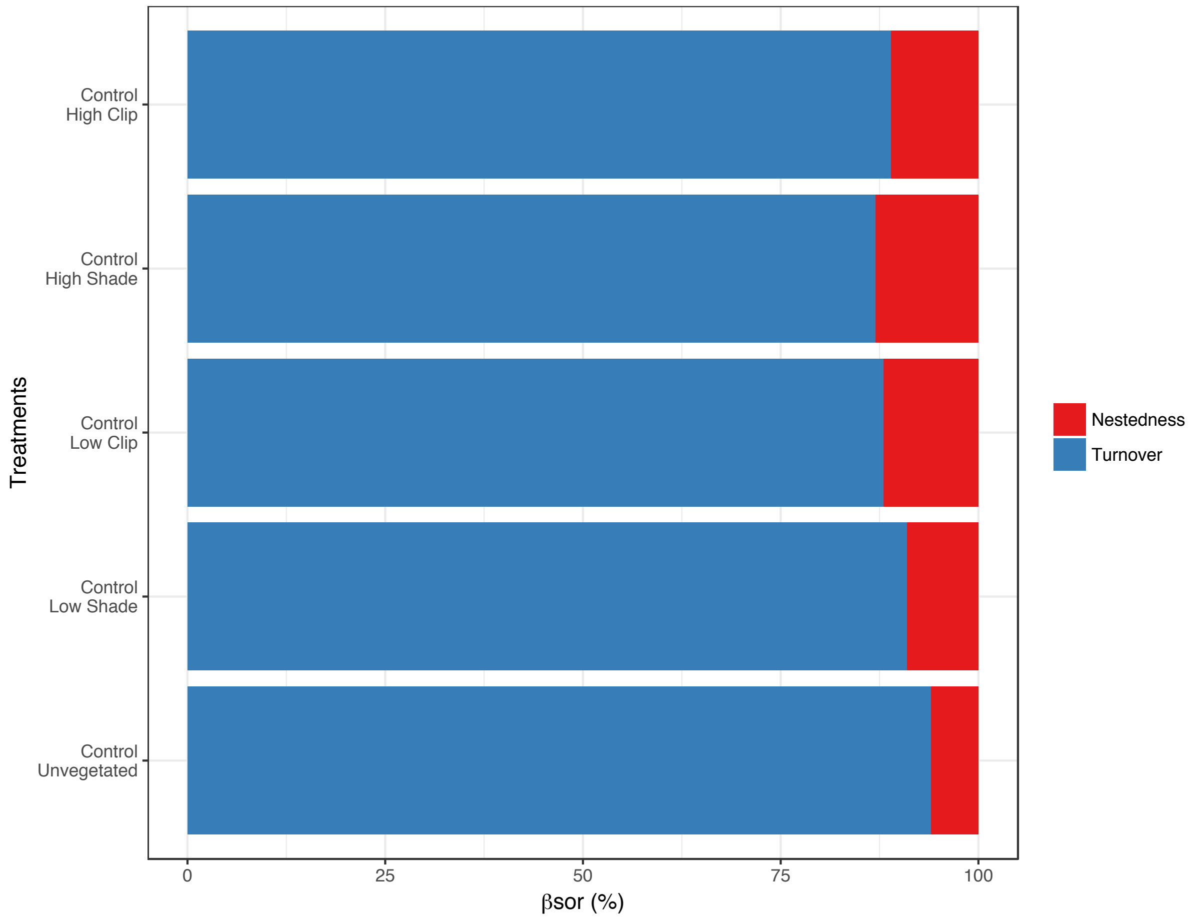

Supplementary Figure 4- Partitioning of beta-diversity between each of the manipulated treatments and the CTRL based on the Sørenson dissimilarity index. Beta-diversity is portioned into dissimilarity due to turnover (Blue) and nestedness (Red).

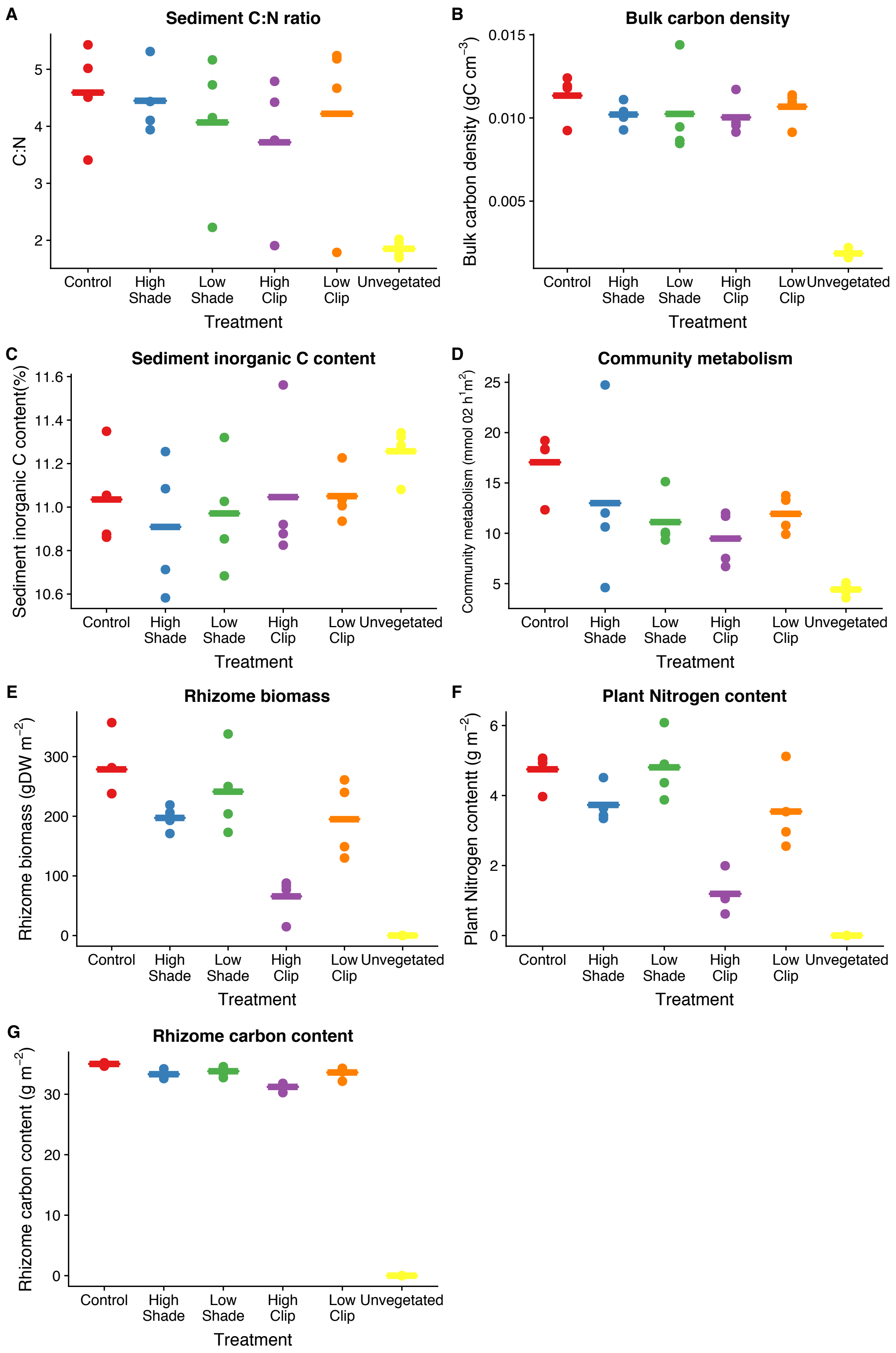
A
Sediment C:N ratio
C:N
Treatment
B
Bulk carbon density
Bulk carbon density (gC cm^{-3})
C
Sediment inorganic C content
Sediment inorganic C content(%)
D
Community metabolism
Community metabolism (mmol 02 h^{1} m^{2})
E
Rhizome biomass
Rhizome biomass (gDW m^{-2})
F
Plant Nitrogen content
Plant Nitrogen contentt (g m^{-2})
G
Rhizome carbon content
Rhizome carbon content (g m^{-2})
Control
High Shade
Low Shade
High Clip
Low Clip
Unvegetated

Supplementary Figure 5- Variation among treatments of the seven environmental variables that compose the combination with the highest correlation to meiobenthos community composition (BIOENV, see Table 1).Figure panels show: Sediment C:N ration (A), Bulk carbon density (B) Sediment inorganic C content (C), Community metabolism (D), Rhizome biomass (E), Nitrogen content in Plant (F), Rhizome content in C (G). Central bars represent the mean of each treatment.
