# Supplementary material for: Above-below surface interactions mediate effects of seagrass disturbance on meiobenthic diversity, nematode and polychaete trophic structure
Source: Commun Biol. 2019 Oct 4;2:362. doi: 10.1038/s42003-019-0610-4 (PMC6778119; doi:10.1038/s42003-019-0610-4)
Supplement: Supplementary file 2 — Description of Additional Supplementary Files [file 42003_2019_610_MOESM2_ESM.docx]

**Description of additional supplementary items**

Supplementary Data 1 - List of all OTUs used in our data analysis including its taxonomic classifications and sequence counts.

Supplementary Data 2 - List classifications of nematode feeding type for the genera investigated in this experiment

Supplementary Data 3 - Complete statistical analyses outputs of the tests presented in the Results and described in the Methods sections
