# Supplementary material for: Above-below surface interactions mediate effects of seagrass disturbance on meiobenthic diversity, nematode and polychaete trophic structure
Source: Commun Biol. 2019 Oct 4;2:362. doi: 10.1038/s42003-019-0610-4 (PMC6778119; doi:10.1038/s42003-019-0610-4)
Supplement: Supplementary file 5 — Supplementary Data 3 [file 42003_2019_610_MOESM5_ESM.pdf]

Differences among treatments in relative abundances between meiobenthic groups

Tested with PERMANOVA

Nematoda

PERMANOVA Summary

Permutation N:	9999
Total sum of squares	8696
Within-group sum of squares	1787
F:	13.92
p (same):	0.0001

PAIRWISE COMPARISIONS WITH CTRLS

p-value

	LowShade	LowClip	HighShade	HighClip	UNV
Control	0.1667	0.1157	0.1117	0.0274	0.03

Copepoda

PERMANOVA Summary

Permutation N:	9999
Total sum of squares	3398
Within-group sum of squares	1442
F:	4.88
p (same):	0.004

PAIRWISE COMPARISIONS WITH CTRLS

p-value

	LowShade	LowClip	HighShade	HighClip	UNV
Control	0.3403	0.0845	0.7385	0.0585	0.0272

Platyhelmintha

PERMANOVA Summary

Permutation N:	9999
Total sum of squares	259.3
Within-group sum of squares	146.3
F:	2.78
p (same):	0.0581

Gastropoda

PERMANOVA Summary

Permutation N:	9999

Total sum of squares	2.148
Within-group sum of squares	1.452
F:	1.726
p (same):	0.1766

Mollusca

PERMANOVA Summary

Permutation N:	9999
Total sum of squares	448.8
Within-group sum of squares	356.6
F:	0.9314
p (same):	0.2533

Polychaeta

PERMANOVA Summary

Permutation N:	9999
Total sum of squares	834.2
Within-group sum of squares	642.3
F:	1.075
p (same):	0.4055

Other

PERMANOVA Summary

Permutation N:	9999
Total sum of squares	4211
Within-group sum of squares	2533
F:	2.384
p (same):	0.0823

Differences among treatments in relative abundances between the most abundant nematode order

Tested with PERMANOVA

Monhysterida

PERMANOVA Summary

Permutation N:	9999
Total sum of squares	0.378
Within-group sum of squares	0.1115
F:	8.611
p (same):	0.0006

PAIRWISE COMPARISIONS WITH CTRLS

	LowShade	LowClip	HighShade	HighClip
Control	0.2048	0.7213	0.972	0.8633

Chromadorida

PERMANOVA Summary

Permutation N:	9999
Total sum of squares	0.01794
Within-group sum of squares	0.007605
F:	4.891
p (same):	0.003

PAIRWISE COMPARISIONS WITH CTRLS

	LowShade	LowClip	HighShade	HighClip
Control	0.1742	0.4856	0.2664	0.7748

Desmodorida

PERMANOVA Summary

Permutation N:	9999
Total sum of squares	0.5143
Within-group sum of squares	0.05346
F:	31.04
p (same):	0.0002

PAIRWISE COMPARISIONS WITH CTRLS

	LowShade	LowClip	HighShade	HighClip
Control	0.0276	0.3641	0.5939	0.5128

Differences among treatments in relative abundances between the most abundant nematode genus

Tested with PERMANOVA

Molgolaimus

PERMANOVA Summary

Permutation N:	9999
Total sum of squares	17.94
Within-group sum of squares	6.654
F:	6.104
p (same):	0.002

PAIRWISE COMPARISIONS WITH CTRLS

	LowShade	LowClip	HighShade	HighClip	UNV
Control	0.9407	0.9402	0.9124	0.7803	0.0302

Monhysteridae

PERMANOVA Summary

Permutation N:	9999
Total sum of squares	71.51
Within-group sum of squares	7.907
F:	28.96
p (same):	0.0001

PAIRWISE COMPARISIONS WITH CTRLS

	LowShade	LowClip	HighShade	HighClip	UNV
Control	0.1725	0.083	0.0902	0.1818	0.0294

Catanema

PERMANOVA Summary

Permutation N:	9999
Total sum of squares	30.26
Within-group sum of squares	1.604
F:	64.3
p (same):	0.0004

PAIRWISE COMPARISIONS WITH CTRLS

	LowShade	LowClip	HighShade	HighClip	UNV
Control	0.828	0.6902	0.8262	0.9124	0.0283

Differences among treatments in relative abundances between the most abundant nematode genus

Tested with PERMANOVA

Observed OTUs

PERMANOVA Summary

Permutation N:	9999
Total sum of squares	8.22E+06
Within-group sum of squares	3.94E+06
F:	3.915
p (same):	0.0131

PAIRWISE COMPARISIONS WITH CTRLS

	LowShade	LowClip	HighShade	HighClip	Unvegetated
Control	0.3952	0.2589	0.1147	1	0.03

ACE

PERMANOVA Summary

Permutation N:	9999
Total sum of squares	1.95E+07
Within-group sum of squares	8.33E+06
F:	4.826
p (same):	0.003

PAIRWISE COMPARISIONS WITH CTRLS

	LowShade	LowClip	HighShade	HighClip	Unvegetated
Control	0.5134	0.4659	0.1185	0.888	0.0309

Shannon

PERMANOVA Summary

Permutation N:	9999
Total sum of squares	4.771
Within-group sum of squares	2.086
F:	4.634
p (same):	0.0104

PAIRWISE COMPARISIONS WITH CTRLS					
	LowShade	LowClip	HighShade	HighClip	Unvegetated
Control	0.6634	0.9718	0.743	0.7366	0.0563

Differences among treatments in meiobenthic community composition

PERMANOVA Adonis Summary

Permutation: free
Number of permutations: 999

	Df	SumOfSqs	R2	F	Pr(>F)
Treatment	5	2.89	0.36	2.04	0.001
Residual	18	5.1	0.64		
Total	23	7.97	1		

PAIRWISE COMPARISIONS WITH CTRLS (pairwise.perm.manova function)

	HighClip	HighShade	LowClip	LowShade	UNV
Control	0.09	0.02	0.04	0.051	0.033

Differences among treatments in meiobenthic community beta-diversity
Tested with betadisp function

PERMANOVA Summary
Permutation: free
Number of permutations: 999

	Df	SumOfSq	Mean Sq	F	Pr(>F)
Treatment	5	4.34E-06	8.69E-07	2.43678	0.0379*
Residuals	18	5.61E-06	3.11E-07		

PAIRWISE COMPARISIC WITH CTRLS

	HighShade	LowShade	HighClip	LowClip	UNV
Control	0.01552562	0.02004538	0.73620275	0.00071107	0.03635139

CCA analysis relating retained environmental variables with meiobenthic community composition

***VECTORS

	CCA1	CCA2	r2	Pr(>r)	
Mean.CN	-0.06701	0.7141	0.7897	0.001	***
MeanBulkC	-0.67191	-0.00269	0.2347	0.31	
Comm_metab	-0.55653	-0.75899	0.3781	0.158	
N_Plant	-0.84846	-0.11493	0.6672	0.004	**
C_Rhizomes	-0.41151	-0.67871	0.7638	0.001	***
MeanIC	-0.20405	-0.2811	0.058	0.85	
