# Supplementary material for: Above-below surface interactions mediate effects of seagrass disturbance on meiobenthic diversity, nematode and polychaete trophic structure
Source: Commun Biol. 2019 Oct 4;2:362. doi: 10.1038/s42003-019-0610-4 (PMC6778119; doi:10.1038/s42003-019-0610-4)
Supplement: Supplementary file 6 — Reporting Summary [file 42003_2019_610_MOESM6_ESM.pdf]

nature research

Corresponding author(s): Francisco J.A. Nascimento

Last updated by author(s): Aug 30, 2019

# Reporting Summary

Nature Research wishes to improve the reproducibility of the work that we publish. This form provides structure for consistency and transparency in reporting. For further information on Nature Research policies, see Authors & Referees and the Editorial Policy Checklist .

## Statistics

For all statistical analyses, confirm that the following items are present in the figure legend, table legend, main text, or Methods section.

| n/a | Confirmed | |
|---|---|---|
| ☐ | ☒ | The exact sample size ($n$) for each experimental group/condition, given as a discrete number and unit of measurement |
| ☐ | ☒ | A statement on whether measurements were taken from distinct samples or whether the same sample was measured repeatedly |
| ☐ | ☒ | The statistical test(s) used AND whether they are one- or two-sided<br>*Only common tests should be described solely by name; describe more complex techniques in the Methods section.* |
| ☐ | ☒ | A description of all covariates tested |
| ☐ | ☒ | A description of any assumptions or corrections, such as tests of normality and adjustment for multiple comparisons |
| ☐ | ☒ | A full description of the statistical parameters including central tendency (e.g. means) or other basic estimates (e.g. regression coefficient) AND variation (e.g. standard deviation) or associated estimates of uncertainty (e.g. confidence intervals) |
| ☐ | ☒ | For null hypothesis testing, the test statistic (e.g. $F$, $t$, $r$) with confidence intervals, effect sizes, degrees of freedom and $P$ value noted<br>*Give P values as exact values whenever suitable.* |
| ☒ | ☐ | For Bayesian analysis, information on the choice of priors and Markov chain Monte Carlo settings |
| ☒ | ☐ | For hierarchical and complex designs, identification of the appropriate level for tests and full reporting of outcomes |
| ☒ | ☐ | Estimates of effect sizes (e.g. Cohen's $d$, Pearson's $r$), indicating how they were calculated |

*Our web collection on statistics for biologists contains articles on many of the points above.*

## Software and code

Policy information about availability of computer code

| | |
|---|---|
| Data collection | No software was used for data collection |
| Data analysis | All statistical tests were performed with R v 3.4.3 |

For manuscripts utilizing custom algorithms or software that are central to the research but not yet described in published literature, software must be made available to editors/reviewers. We strongly encourage code deposition in a community repository (e.g. GitHub). See the Nature Research guidelines for submitting code & software for further information.

## Data

Policy information about availability of data

All manuscripts must include a data availability statement. This statement should provide the following information, where applicable:

- Accession codes, unique identifiers, or web links for publicly available datasets
- A list of figures that have associated raw data
- A description of any restrictions on data availability

The raw sequence data have been uploaded and are available on the NCBI database with the following BioProject number: PRJNA540961

# Field-specific reporting

Please select the one below that is the best fit for your research. If you are not sure, read the appropriate sections before making your selection.

☐ Life sciences ☐ Behavioural & social sciences ☒ Ecological, evolutionary & environmental sciences

For a reference copy of the document with all sections, see nature.com/documents/nr-reporting-summary-flat.pdf

# Ecological, evolutionary & environmental sciences study design

All studies must disclose on these points even when the disclosure is negative.

| | |
|---|---|
| Study description | Effects of seagrass disturbance on meiobenthos. In situ factorial experiment with 6 treatments, 4 replicates each |
| Research sample | Benthic meiofaunal community isolated on a 40 micron sieve, then extracted from sediment particles with density extraction |
| Sampling strategy | Plots were placed within a 40 x 40 m experimental site using a random block design, with each plot covering 10 m2. Each of the 24 replicate plot was sampled with six handheld Perspex sediment cores taken from the same points inside the plots |
| Data collection | The top 3 cm of each core were sliced pooled and sieved through 500 micron and 40 microns stacked sieves and preserved. This was done by the authors |
| Timing and spatial scale | Sampling was done once  5 months after the start of the experiment. The experimental site covered an area of 40x40m |
| Data exclusions | Data analyses focused on metazoan meiofauna. Community composition was examined by filtering out non-metazoan eukaryotic OTUs as they were not the focus of our stud |
| Reproducibility | The experimental procedures are described with enough detail to allow for reproducibility |
| Randomization | we used a random block design (Fig.7)  and randomized the order of all sample analysis |
| Blinding | No binding necessary in our study |

Did the study involve field work? ☒ Yes ☐ No

## Field work, collection and transport

| | |
|---|---|
| Field conditions | Experiment done semi-enclosed bay on the east coast of Zanzibar Island with a maximum (spring tide) tidal fluctuation of 3.2 m. The bay is dominated by seagrass meadows (with as many as 11 seagrass species) and unvegetated bare sediment habitats |
| Location | Seagrass meadow in Chwaka Bay on Zanzibar Island (Unguja), Tanzania. Coordinates: 06°09'S 39°26'E |
| Access and import/export | Experiment was done together with Dodoma University, Tanzania, and followed, local, national and international regulations |
| Disturbance | We simulated disturbance in the experimental plots (shading and removal of biomass). Disturbance of the areas outside the experiment were minimal |

# Reporting for specific materials, systems and methods

We require information from authors about some types of materials, experimental systems and methods used in many studies. Here, indicate whether each material, system or method listed is relevant to your study. If you are not sure if a list item applies to your research, read the appropriate section before selecting a response.

## Materials & experimental systems

| n/a | Involved in the study | |
|---|---|---|
| ☒ | ☐ | Antibodies |
| ☒ | ☐ | Eukaryotic cell lines |
| ☒ | ☐ | Palaeontology |
| ☐ | ☒ | Animals and other organisms |
| ☒ | ☐ | Human research participants |
| ☒ | ☐ | Clinical data |

## Methods

| n/a | Involved in the study | |
|---|---|---|
| ☒ | ☐ | ChIP-seq |
| ☒ | ☐ | Flow cytometry |
| ☒ | ☐ | MRI-based neuroimaging |

## Animals and other organisms

Policy information about studies involving animals; ARRIVE guidelines recommended for reporting animal research

| | |
|---|---|
| Laboratory animals | No laboratory animals were used in our study |
| Wild animals | We sampled only microscopic invertebrate animals |
| Field-collected samples | We collected microscopic invertebrate animals |

| Ethics oversight | No ethical approval was required |
| --- | --- |

Note that full information on the approval of the study protocol must also be provided in the manuscript.
